# Draft genome of the river water buffalo

**DOI:** 10.1002/ece3.4965

**Published:** 2019-02-18

**Authors:** Abdul Awal Mintoo, Hailin Zhang, Chunhai Chen, Mohammad Moniruzzaman, Tingxian Deng, Mahbub Anam, Quazi Mohammad Emdadul Huque, Xuanmin Guang, Ping Wang, Zhen Zhong, Pengfei Han, Asma Khatun, Tabith M. Awal, Qiang Gao, Xianwei Liang

**Affiliations:** ^1^ Lal Teer Seed Limited Dhaka Bangladesh; ^2^ Lal Teer Livestock Limited Dhaka Bangladesh; ^3^ MNT Life Sciences Center Gazipur Bangladesh; ^4^ BGI‐Genomics, BGI‐Shenzhen Shenzhen China; ^5^ BGI Education Center University of Chinese Academy of Sciences Beijing China; ^6^ Key Laboratory of Buffalo Genetics, Breeding and Reproduction Technology, Buffalo Research Institute Chinese Academy of Agricultural Sciences Nanning China

**Keywords:** annotation, genome, phylogenetic analysis, water buffalo

## Abstract

Water buffalo (*Bubalus bubalis*), a large‐sized member of the Bovidae family, is considered as an important livestock species throughout Southeast Asia. In order to better understand the molecular basis of buffalo improvement and breeding, we sequenced and assembled the genome (2n=50) of a river buffalo species *Bubalus bubalis *from Bangladesh. Its genome size is 2.77 Gb, with a contig N50 of 25 kb and the scaffold N50 of 6.9 Mbp. Based on the assembled genome, we annotated 24,613 genes for future functional genomics studies. Phylogenetic tree analysis of cattle and water buffalo lineages showed that they diverged about 5.8–9.8 million years ago. Our findings provide an insight into the water buffalo genome which will contribute in further research on buffalo such as molecular breeding, understanding complex traits, conservation, and biodiversity.

## 1 BACKGROUND

Water buffalo was domesticated over 3,000–6,000 years ago and are economically important animals in many parts of the world. They provide more than 5% world's milk supply. Their milk has higher fat, lactose, protein, and higher minerals content than the milk of the cow. Buffalo milk is used to make butter, butter oil, high quality cheeses, and various other higher quality dairy products (Buffalo, 2000; Roth & Myers, 2004). Their meat is very tender and palatable and their hides have economic importance as the raw materials of high quality leather products.

In many parts of the world, especially in Southeast Asia countries, water buffalo provides 20%–30% of farm power, and their dung is used as fertilizer and fuel in many highly populated countries (Bilal, Suleman, & Raziq, 2006; Dixon et al., 2001; Kierstein et al., 2004; Yindee et al., 2010) There are two types of domestic water buffalo, the River buffalo (*Bubalus bubalis*, 2n=50) and the Swamp Buffalo (*Bubalus carabanesis*, 2n=48).These two types were distinguished by the Karyotypes (Michelizzi et al., 2010). Swamp type is widely used for draught, but river type is mostly used for milk and meat. It is known that river type buffalo is the second largest dairy species in the world. In addition to milk, a significant amount of Asian meat and hide production comes from buffalo (Pasha & Hayat, 2012). Buffalo meat is healthier as it is relatively lean with a fat content of less than 2% (Borghese, & Manzi, 2005).

Limited research has been conducted globally in order to explore the genetic diversity, and molecular genetic basis in buffalo compared to other farm animals (Gonçalves, Silva, Barbosa, & Schneider, 2004). Molecular markers can be a powerful tool for livestock improvement through breeding strategies. Based on the cattle genome, Madhu et al. assembled the buffalo genome with 17–19× Illumina reads and only with a median contig length of 2.3 kb (Tantia et al., 2011). As poorly assembled results, they only identified some SNPs and indels in the buffalo genome (Tantia et al., 2011). Recently, Glanzmann et al. (2016) reported the African buffalo genome (*Syncerus caffer,* 2n=52) which was assembled as 2.68 Gb with a contig N50 of 43 kbp and Scaffold N50 of 2.3 Mbp. Genome analysis has found 19,765 genes and 97.60% of them could be successfully annotated. Moreover, they identified some expanded predicted genes which are coupled to a large variety of GO terms, including G‐coupled protein and olfactory receptors. Williams et al. (2017) assembled the water buffalo genome and obtained 2.83 Gb size with the scaffold N50 of 1.4 Mb and contig N50 of 21.4 kb. Based on the RNA‐seq data, they identified 21,711 protein coding genes. Although they presented the water buffalo genome, the completeness of the genome such as the scaffold N50 can still be improved. In this study, we present sequencing and de novo genome assembly of Bangladeshi buffalo which is a river type water buffalo. The results obtained in this study could be used for the breed development of water buffalo through molecular breeding not only in productive traits to ensure higher milk and meat yield but also disease resistance and environmental adaptivity in the changing global climate.

## 2 MATERIALS AND METHODS

### 2.1 Genomic DNA and sequencing

The river water buffalo genomic DNA was extracted from an eight‐year‐old plain land reverian type male water buffalo's blood from Bangladesh. A series of short‐insert (170, 500, and 800 bp) DNA libraries were constructed according to the Illumina manufacturer's protocol. As for the long‐insert mate‐paired libraries (>1 kb), approximately 20–50 μg genomic DNA was fragmented, biotin labeled, circularized, broken, and enriched using biotin/streptavidin, to generate the libraries. Illumina HiSeq2000 paired‐end sequencing was performed with PE101 for short‐insert libraries, and PE50 for long‐insert libraries.

### 2.2 Data filtering and error correction

The raw reads 350.80 Gbp generated from the Solexa‐Pipeline should be filtered. The filtering criteria were as follows: (a) filtered reads with more than 2% of Ns or containing polyA structure; (b) filtered reads where 40% of the bases had a low Phred quality values (<8) for short‐insert libraries and 60% bases for large‐insert libraries; (c) filtered reads with more than 10 bp aligned to the adapter sequence (allowing <4 bp mismatch); (d) filtered paired reads with overlapped sequence length >10 bp (allowing 10% mismatch); (e) filtered PCR‐duplicated reads. The filtered reads is 255.95 Gb.

We built a K‐mer (*K* = 17) frequency table, set the cutoff at 8 for dividing low‐frequency K‐mers and high‐frequency ones, and performed the error correction step to eliminate sequencing errors. Low‐frequency K‐mers were corrected in the error bases or trimmed at the ends to form high‐frequency K‐mers.

### 2.3 Genome assembly

Based on SOAPdenovo V2.01 (Luo et al., 2012), the error‐corrected short‐insert library reads were split into K‐mers (*K* = 41) to first construct a de Bruijn graph, which was simplified by removing tips, merging bubbles, and solving repeats to get 235,999 contigs. Secondly, we realigned all reads gradually to the contig sequences to determine the shared paired‐end relationships, weigh the rate of consistent and conflicting paired ends, and construct 33,840 scaffolds. Also, we utilized SSPACE V1.1 (Boetzer, Henkel, Jansen, Butler, & Pirovano, 2010) to improve scaffold lengths. Finally, we extracted the short‐insert size reads with one end mapped to the contig and the other end located in the inner‐scaffold gap region, then we performed a local assembly to fill the gaps.

### 2.4 CpG island identification

We identified CpG islands in the water buffalo and cattle genomes by using the search algorithm developed by Takai and Jones (Takai & Jones, 2002). Regions of DNA of greater than 500 bp with a G + C equal to or greater than 55% and observed CpG/expected CpG of 0.65 were more likely to be associated with the 5’ regions of genes and this definition excludes most Alu‐repetitive elements.

### 2.5 Identification of synteny and segmental duplication

With parameters *T* = 2, *C* = 2, *H* = 2000, *Y* = 3,400, *L* = 6,000, and *K* = 2,200, we used LASTZ (Harris, 2007) to detect synteny blocks between the water buffalo and other mammals. Before the genome alignment, we downloaded the masked repeat genome of cattle from ensemble and used the following method to mask the buffalo genome, after that LASTZ was used for alignment. Based on the self‐alignment implement, we used whole‐genome assembly comparison (WGAC) to identify segmental duplications by LASTZ. We defined segmental duplications to be two sequences with a length larger than 1 kb and have a higher identity than 90% and lower than 99.5%. The resulting alignments that extended to >1 kb length and had >90% sequence identity were deemed recent segmental duplications.

### 2.6 Annotation of repeats and non‐coding RNA

We identified repeat sequences by combining the homologous strategy and de novo method. Based on the homologous strategy, known transposable elements (TEs) were identified against the Repbase (Jurka et al., 2005) 16.10 TE library using RepeatMasker (Version 3.3.0) (Smit et al.) and RepeatProteinMask at the DNA and protein level. In parallel, we annotated the tandem repeats using Tandem Repeats Finder (Version 4.04) (Benson, 1999). To generate a de novo repeat library, we implemented the LTR*_*finder (Version 1.05) (Xu & Wang, 2007), Repeatmodeler (Version 1.0.5) (Smit & Hubley), and a RepeatMasker analysis. We detected four types of non‐coding RNAs by searching the whole‐genome sequence. The transfer RNAs were predicted by tRNAscan‐SE‐1.23(Lowe & Eddy, 1997). The ribosomal RNAs were found by aligning with the human rRNA sequences. The snRNAs and miRNAs were identified by aligning with BLASTN (Version 2.2.23) (Altschul et al., 1997) and then searched against the Rfam (Version 9.1) (Griffiths‐Jones et al., 2005).

### 2.7 Gene prediction

Three basic approaches, such as de novo, homology‐based and RNA‐seq methods, were utilized to predict gene structures. With the help of AUGUSTUS (Stanke et al., 2006) and GENESCAN (Burge & Karlin, 1997), we performed the de novo prediction with the foundation of the repeat‐masked genome. Using TBLASTN (Altschul et al., 1997) with an *E*‐value cutoff 1*e*−5, we mapped several homologous proteins of following mammalian species to the water buffalo genome: *Bison bonasus* (NCBI), *Bubalus bubalis *(NCBI), *Bos grunniens *(Ensembl), *Bos taurus *(Ensembl), *Equus caballus *(Ensembl), *Homo sapiens *(Ensembl), and *Ovis aries *(Ensembl) (Aken et al., 2016; Wheeler et al., 2007). Then, the aligned sequences were filtered by Solar (Li et al., 2010) (Version 0.9.6) and passed to GENEWISE (Birney, Clamp, & Durbin, 2004) to search for accurately spliced alignments. For the RNA‐seq based prediction, transcriptome reads (SRR527267, SRR527268, SRR527269, SRR527270) were mapped to the genome using HISAT (hisat2‐2.0.1‐beta) (Kim, Langmead, & Salzberg, 2015). Then, we combined HISAT mapping results together and applied StringTie (Pertea et al., 2015) to predict transcript structures. Eventually, a consensus gene set was produced using EVM (Haas et al., 2008) to integrate the source evidence generated from both approaches above.

### 2.8 Function annotation

Based on the databases TrEMBL (Bairoch & Apweiler, 2000) and SwissProt (Bairoch & Apweiler, 2000), we assigned gene functions in accordance with the best match of the alignments. The domains and motifs of water buffalo genes were acknowledged by InterProScan (Quevillon et al., 2005) against six protein databases, including ProDom, Pfam, PRINTS, PANTHER, PROSITE, and SMART. Meanwhile, we obtained Gene Ontology (GO) (Ashburner et al., 2000) IDs for each gene from the corresponding InterPro entries. At last, we aligned all genes against KEGG proteins and pathway.

### 2.9 Gene family clusters

We used the Treefam (Li et al., 2006) methodology to define a gene family as a group of genes that descended from a single gene in the last common ancestor of a considered species. As per the following steps: (a) Blastp was applied to all protein sequences (water buffalo, Bactrian Camel, cattle, horse, yak, American bison) against a database containing a protein dataset of all species with the *e*‐value of 1 × *e* ^−7^ and conjoined fragmental alignments for each gene pair by Solar (Li et al., 2010). We assigned a connection (edge) between the two nodes (genes) if more than one‐third of the region aligned to both genes. An H‐score that ranged from 0 to 100 was used to weigh the similarity (edge). (b) Extraction of gene families (clustering by *H‐cluster_sg*). We used the average distance for the hierarchical clustering algorithm, requiring the minimum edge weight (H‐score) to be larger than 5, and the minimum edge density (total number of edges/theoretical number of edges) to be larger than one‐third.

### 2.10 Gene family expansion and contraction

We used CAFÉ (De Bie, Cristianini, Demuth, & Hahn, 2006) to analyze changes in gene family size under a random birth and death model, over the phylogenetic tree with divergence times. A global gene birth and death rate parameter *λ* across all branches for all gene families was estimated using the maximum likelihood method. The conditional *p*‐value was calculated, and families with a *p*‐value <0.05 were determined to suffer significant family changes.

### 2.11 Divergence time

The CDS sequences of the single‐copy orthologous genes were used for estimating divergence times based on the phylogenetic tree. The PAML (Yang, 2007) MCMCTREE (Yang & Rannala, 2006) performs Bayesian estimation of species divergence times using soft fossil constraints under various molecular clock models. The Markov chain Monte Carlo (MCMC) process of PAML MCMCTREE was run to sample 1,000,000 times, with sample frequency set to 50, after a burn‐in of 5,000,000 iterations. Parameters of “finetune” were set as “0.004, 0.016, 0.01, 0.10, 0.58.” Other parameters were set as default. We did two independent runs to confirm that the different runs produced very similar results.

### 2.12 Positive selection analysis

As mentioned above, we obtained single‐copy orthologous genes from six species. Then, we calculated dN/dS ratios for these single‐copy orthologous genes using codeml in the PAML (Yang, 2007) package to estimate positive selection. Then, we used PRANK (Löytynoja & Goldman, 2008) and Gblocks (Castresana, 2000) software to estimate Ka, Ks, and Ka/Ks ratios on each branch.

### 2.13 Demographic history

SNPs of the sequenced buffalo were used to reconstruct demographic history with the PSMC model (Li & Durbin, 2011) with the generation time (*g* = 3) and mutation rates (*μ* = 2.5 × 10^−8^). Parameters were set as follows: −N 25, −t 15, −r 5 − p “4 + 25*2 + 4 + 6”. Following Li's procedure, we applied a bootstrapping approach, repeating sampling 100 times to estimate the variance of simulated results.

## 3 RESULT AND DISCUSSION

**Figure 1:**
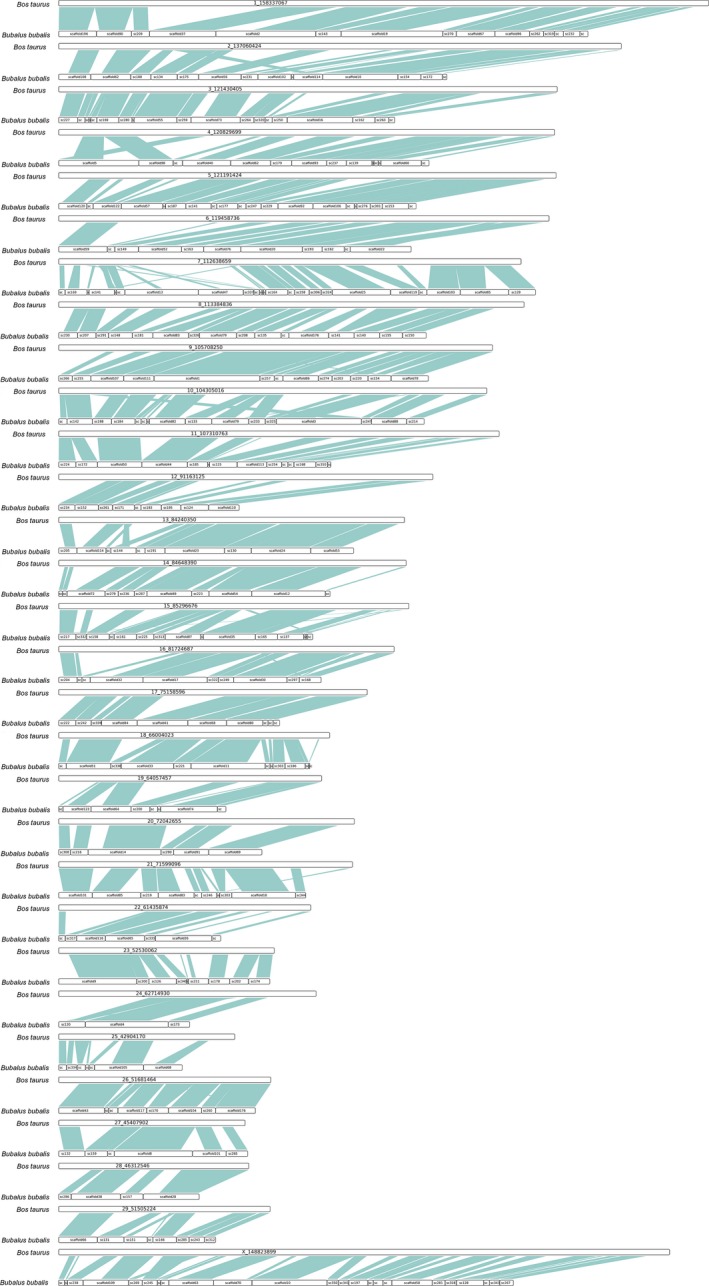
The synteny block between the genome of water buffalo and cow

Libraries were constructed with insert sizes ranging from 170 bp to 20 kbp, from which 350.80Gbp of paired‐end sequencing data were generated using the Illumina Hiseq2000 platform. After filtering out low quality, adapter‐contaminated, PCR‐duplicated, and small‐insert reads, we obtained 255.95 Gbp of clean data, covering the water buffalo genome with an approximately 87‐fold depth and 2,777‐fold physical depth (Table 1). The water buffalo genome size was estimated to be 2.95 Gbp. After the error‐containing library data with low‐frequency K‐mers of short insert size (<1 kbp) had been corrected, contigs and scaffolds were constructed using the data with SOAPdenovo software, further super scaffolds were built by *SSPACE*, and the inner‐scaffold gaps were filled by GapCloser. A total of 2.77 Gbp of assembled sequences were obtained, with a contig N50 of 25 kbp and scaffold N50 of 6.96 Mbp. These assembled results were comparable to those of the previously obtained African buffalo genome and water buffalo genome (Table 2). In order to evaluate the genome completeness, two different methods were used. The first one was based on EST/mRNA sequences downloaded from the NCBI and aligned to our genome by BLAT (Kent, 2002), where approximately 98.15% of the data could be well aligned, demonstrating a well‐assembled genome. The second method involved benchmarking against universal single‐copy orthologs (BUSCO 2.0), where our assembly covered 94.3% of the core genes, with 3,870 genes being completed. This also implied the high quality of our assembly. We identified more CpG islands in the water buffalo genome (39,578) than in the cattle genome (12,120) (Han, Su, Li, & Zhao, 2008). This difference was mainly because these two species have different recombination rate and chromosome size (Jobse et al., 1995). On the basis of LASTZ alignment, we identified a syntenic region of approximately 2,322 Mbp between the water buffalo and cattle genomes, with a coverage rate of 83.31%. The syntenic region included 15,361 coding genes (Figure 1). Moreover, we estimated the segmental duplication of the buffalo genome and found a 94.5 Mbp length that was comparable to that previous report for cattle (94.4 Mbp) (Elsik, Tellam, & Worley, 2009). The similar segmental duplication lengths could mean that the duplication events had occurred in the last common ancestor of the water buffalo and cattle (Li et al., 2018). To determine the sequence difference between the two species, copy number variations (CNVs) were used as the finder to detect the deletion or duplication in the genomic region. By aligning the reads of the water buffalo to the cattle genome, we identified 16,207 block deletions and 21 Mbp duplication on the basis of BWA (Li & Durbin, 2009), CNVnator (Abyzov, Urban, Snyder, & Gerstein, 2011), and SAMtools (Li et al., 2009). It is possible that the segmental duplications of the water buffalo genome are different from the cattle reference (Table 3).

**Table 1:** The statistics for raw data and clean data

Pair‐end libraries	Insert size	Reads length	Raw data	Clean data		
			Total data (Gb)	Total data (Gb)	Sequence depth (X)	Physical depth (X)
Solexa reads	170 bp	100_100	37.94	31.07	10.55	8.97
	500 bp	100_100	64.89	57.46	19.50	48.76
	800 bp	100_100	44.68	38.38	13.03	52.11
	2 kb	49_49	93.38	66.66	22.63	461.77
	5 kb	49_49	40.43	25.55	8.67	442.47
	10 kb	49_49	34.18	22.78	7.73	788.90
	20 kb	49_49	35.30	14.05	4.77	973.60
Total	—	—	350.80	255.95	86.88	2,776.57

Note

Assuming the genome size is 2.946 Gb.

**Table 2:** Assembly statistics of our River water buffalo genome, African buffalo, and published water buffalo genome

	River Water buffalo	African buffalo	Water Buffalo#
Contig			
N50	25,036	42,601	21,938
Largest	262,402	471,476	—
Number	235,999	561,609	630,368
Scaffold			
N50	6,957,949	2,411,048	1,412,388
Largest	25,744,419	16,927,952	—
Number	33,840	442,401	366,983
Total assembled size (bp)	2,770,477,792	2,688,614,675	2,836,166,969

Note

“Water Buffalo #” was represented the assembly UMD_CASPUR_WB_2.0 from the paper.

**Table 3:** Summary statistics cattle reference CNVs using buffalo reads

Range	Deletion			Duplication		
	Block	Length	Cover	Block	Length	Cover
>1 kb	16,207	3,000	113,985,100	1,475	9,300	20,914,100
>5 kb	5,468	9,700	88,900,300	1,101	12,800	19,716,700
>10 kb	2,601	15,900	68,317,500	689	19,000	16,688,100
>20 kb	907	30,700	45,028,000	316	29,300	11,383,700
>50 kb	235	82,300	25,510,100	47	61,800	3,475,500

The water buffalo contained 1,418 Mbp of repetitive DNA, accounting for 51.19% of the genome, which is comparable to the percentages in humans (46.8%), mice (42.5%), dogs (40.0%), cattle (47.1%), and pigs (39.1%) (Huang et al., 2014). It showed an ~13% higher repeat content relative to that of the African buffalo (37.21%) (Glanzmann et al., 2016). Notably, the content of transposable elements (TEs) in the water buffalo genome was 49.06%, of which long interspersed nuclear elements (LINEs) accounted for 41.50% as the main TE component (Table 4). A similar tandem repeat content of genomes is observed in other mammals, such as human (45%). Moreover, we annotated 23,310 microRNAs (miRNAs), 38,483 transfer RNAs (tRNAs), 867 ribosomal RNAs (rRNAs), and 1,758 small nuclear RNAs (snRNAs) in the water buffalo genome (Table 5).

**Table 4:** Summary statistics of interspersed repeat regions in *Bubalus bubalis*

Type	Repbase TEs		TE proteins		De novo		Combined TEs	
	Length (bp)	% in genome	Length (bp)	% in genome	Length (bp)	% in genome	Length (bp)	% in genome
DNA	36,153,654	1.30	7,072,414	0.26	4,337,092	0.16	40,080,739	1.45
LINE	601,949,239	21.73	395,094,173	14.26	958,438,214	34.60	1,094,513,235	39.51
SINE	201,003,037	7.26	0	0.00	11,615,206	0.42	210,326,622	7.59
LTR	100,299,951	3.62	11,867,770	0.43	296,982,552	10.72	375,094,177	13.54
Other	272	0.00	0	0.00	0	0.00	272	0.00
Unknown	0	0.00	0	0.00	134,207	0.00	134,207	0.00
Total	921,567,446	33.26	413,831,008	14.94	1,118,906,262	40.39	1,255,723,859	45.33

**Table 5:** Summary statistics of non‐coding RNAs in *Bubalus bubalis*

Type	Copy number	Average length (bp)	Total length (bp)	% of genome
miRNA	23,310	100.82	2,350,000	0.0848
tRNA	38,483	72.86	2,803,734	0.1012
rRNA	867	105.79	91,722	0.0033
18S	123	135.18	16,627	0.0006
28S	271	146.65	39,741	0.0014
5.8S	9	81.89	737	0.0000
5S	464	74.61	34,617	0.0013
snRNA	1,762	114.17	201,174	0.0073
CD‐box	319	92.78	29,598	0.0011
HACA‐box	300	135.20	40,560	0.0015
Splicing	1,106	114.34	126,457	0.0046

**Figure 2:**
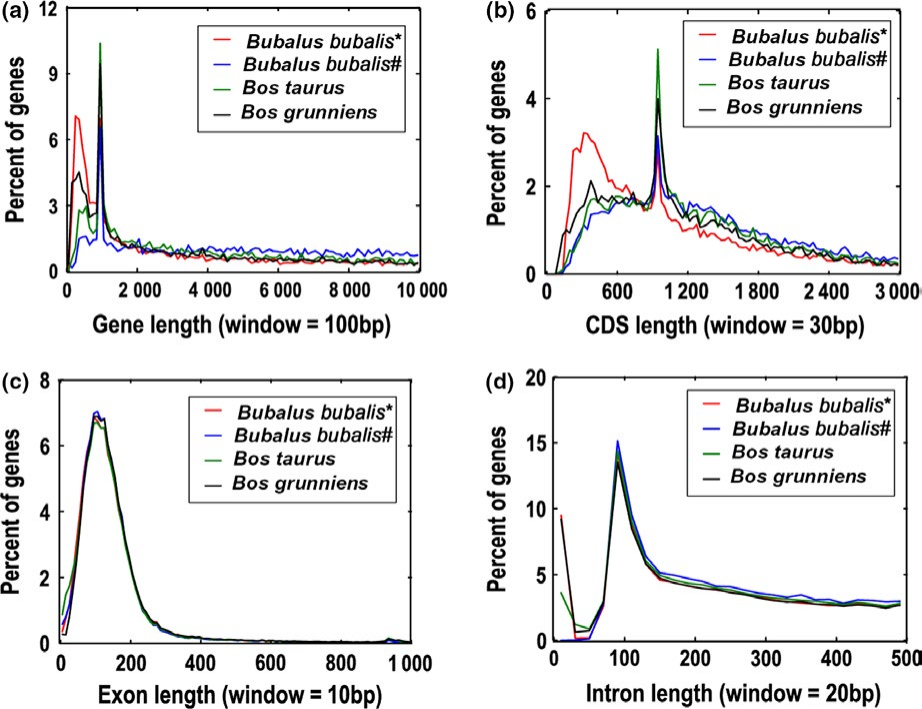
Comparison of gene parameters among the Bovidae family genome. (a) Gene length; (b) CDS length; (c) exon length; (d) intron length

On the basis of a combination of ab initio gene finders, a homology‐based method, and an RNA‐Seq method, we predicted 24,613 water buffalo genes (Table 6). BUSCO (Simão, Waterhouse, Ioannidis, Kriventseva, & Zdobnov, 2015) was carried out to evaluate the gene prediction quality, and results showed that 97.1% of orthologs could be found in our annotation (Table 7), which was suggestive of a complete assembly and annotation. This gene set quality was comparable to those of the *B. taurus *(UMD3.1), *B. bubalis* (UMD_CASPUR_WB_2.0), and *B. grunniens* genomes. Moreover, we compared the length distribution of genes, coding sequences (CDS), exons and introns among human and other mammalian genomes and found similar distribution of these parameters (Figure 2).

**Table 6:** Summary statistics of denovo, homolog, transcript approaches and integrate the gene prediction in *Bubalus bubalis*

Gene set	Number	Average gene length (bp)	Average CDS length (bp)	Average exons per gene	Average exon length (bp)	Average intron length (bp)
AUGUSTUS	21,098	50,022	1,453	9	166.05	6,266
*Bos grunniens*	27,004	21,134	1,272	7	177.59	3,224
*Bubalus bubalis*	25,417	23,299	1,343	7	181.74	3,435
*Bos taurus*	24,332	22,204	1,343	7	181.01	3,250
*Bison bonasus*	24,849	23,684	1,354	7	180.79	3,440
*Ovis aries*	25,515	22,246	1,322	7	181.52	3,330
*Homo sapiens*	26,247	21,689	1,248	7	181.92	3,488
*Equus caballus*	24,378	21,661	1,290	7	183.08	3,368
Transcript	95,359	3,145	893	3	319.30	1,254
Homolog and transcript	34,560	18,446	1,128	6	183.90	3,325
End integrate	24,613	45,255	1,407	9	164.26	5,789

**Table 7:** Summarized benchmarks in the BUSCO assessment for genome assembly and genesets

BUSCO benchmark	B. bubalis* Number/%		*B. bubalis* Number/%		*B. grunniens *Number/%		*B. taurus* Number/%	
	Genesets	Genome	Genesets	Genome	Genesets	Genome	Genesets	Genome
Complete single‐copy	2,395/92.6	3,870/94.3	2,387/92.3	1680/40.9	2,389/92.4	3,987/97.1	2,399/92.8	3,785/92.2
Complete duplicated	39/1.5	37/0.9	42/1.6	750/18.3	29/1.1	27/0.7	44/1.7	248/6
Fragmented	81/3.1	78/1.9	90/3.5	105/2.6	99/3.8	59/1.4	79/3.1	50/1.2
Missing	71/2.8	119/2.9	67/2.6	1569/38.2	69/2.7	31/0.8	64/2.4	21/0.6

Note

“*B. bubalis* *” was studied in this paper. “*B. bubalis* #” represented the assembly UMD_CASPUR_WB_2.0. BUSCO version is: 2.0. The lineage dataset is: vertebrata_odb9 (Creation date: 2016‐02‐13, number of species: 65, number of BUSCOs: 4,041).

**Figure 3:**
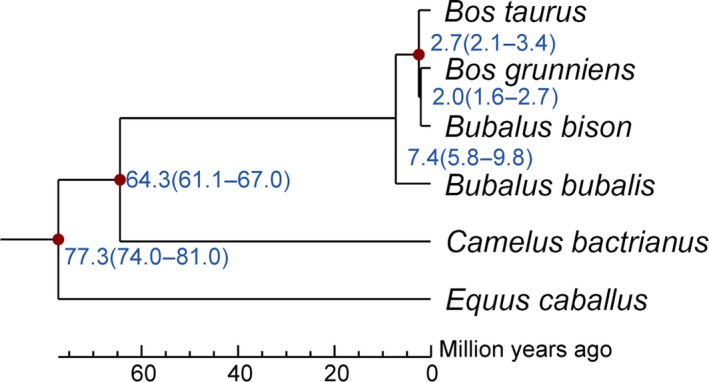
Estimation of divergence time. The numbers on the nodes represent the divergence times from present (million years ago, Mya).The red points in three internal nodes indicate fossil calibration times for *Equus caballus‐Bos taurus *divergence (74–81Mya), *Camelus bactrianus*‐*Bos taurus* divergence (61–71Mya), and *Bos Taurus‐Bos grunniens* divergence (1.96–6.77Mya) (http://www.timetree.org/) used in the analysis. The estimated divergence times with their 95% confidence intervals are shown

TreeFam methodology was used to examine the conservation of gene repertoires among water buffalo genes and five other mammals (viz., horse, yak, Bactrian camel, American bison, cattle). A total of 18,015 water buffalo genes were grouped into 13,985 orthologous families, whereas 238 unique gene families were found. Among these, genes had significant GO enrichment (*p* < 0.001) in the intracellular organelle part (GO:0044446), intracellular organelle (GO:0043229), macromolecular complex (GO:0032991), and scavenger receptor activity (GO:0005044) terms. We constructed a phylogenetic tree via the maximum likelihood method by applying PHyML (Guindon et al., 2010), using 7,090 single‐copy orthologous genes on 4‐fold degenerate sites among mammals under the GTR+gamma model. Analysis based on the same data set dated the most recent common ancestor of the water buffalo and cattle to approximately 5.8–9.8 million years (Figure 3).

Next, we identified 159 gene families that were substantially expanded in the water buffalo compared with other mammals. Functional categories for these significant expanded gene families belonged mainly to signaling receptor activity (GO:0038023, *p* < 0.001), ATP‐binding (GO:0005524, *p* < 0.001), olfactory receptor activity (GO:0004984, *p* < 0.001), G‐protein‐coupled receptor signaling pathway (GO:0007186, *p* < 0.001), inositol 1,4,5‐trisphosphate‐sensitive calcium‐release channel activity (GO:0005220, *p* < 0.001), and transmembrane transport (GO:0055085, *p* < 0.001). It is possible that these expanded genes are related to environmental adaptation and specific molecular genetics mechanisms. To identify genes that might be candidates for the water buffalo's adaption to its environment, we identified 382 genes that contained positive selection sites in buffalo. These genes were mostly annotated to signal transduction pathway, metabolic pathway, and immune system functional pathway.

**Figure 4:**
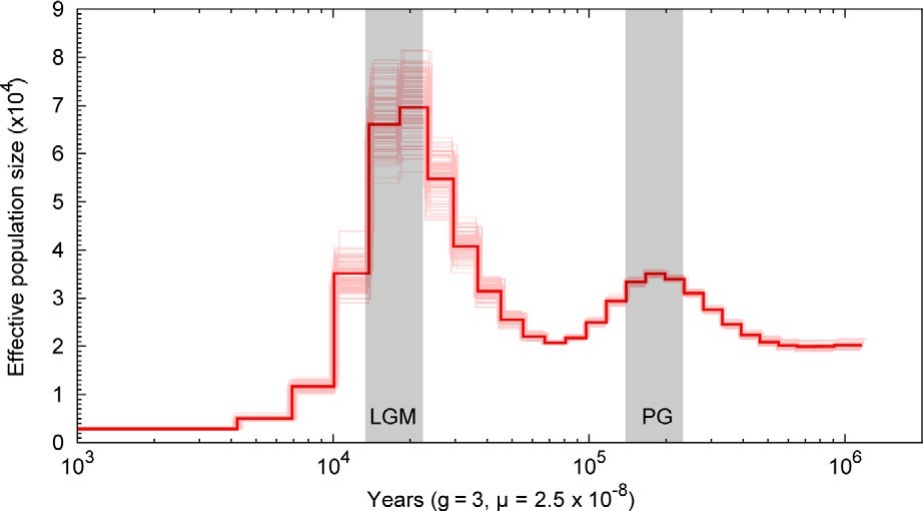
Demographic history inferred from a single buffalo genome. Buffalo populations reached a maximum size coinciding with the largest glacial maximum (LGM) at about 20,000 years ago and rised to another peak almost simultaneous with the Penultimate Glaciation (PG) at about 200,000 years ago (vertical gray shadow on graph). The graph's horizontal axis shows the measurement of time by pairwise sequence divergence, and the vertical axis shows the measurement of the effective population size by the scaled mutation rate. The light pink lines correspond to PSMC inferences on 100 rounds of bootstrapped sequences and the red line stands for the estimate from the data

It is interesting to infer the demography of a diploid species up to hundreds of generations ago using its whole‐genome sequence data (e.g., by using pairwise sequential Markovian coalescence; PSMC) (Li & Durbin, 2011). The reads used for assembling the buffalo genome were mapped onto the assembled genome. A total of 5,704,306 heterozygous loci were identified as putative heterozygotes in the genome with a heterozygosity rate of 0.2% as obtained by BWA (Li & Durbin, 2009) and SAMtools (Li et al., 2009). We employed the PSMC method to explore the changes in effective population size (Ne) of the ancestral population of the buffalo to accommodate the Quaternary climatic change (Kelley et al., 2014). Assuming that the inference of the mutation rate for buffalo is correct, the analysis suggests that the buffalo population expansions occurred before the advent of penultimate glaciation (PG) at about 200,000 years ago and the population size declines after the retreat of PG (Figure 4). When climate became favorable, the buffalo population size had reached a maximum size coinciding with the largest glacial maximum (LGM) at about 20,000 years ago (Zheng, Xu, & Shen, 2002). Subsequently, the buffalo population plummeted in the late period of the LGM, which may have led to the grassland degeneration and forest vegetation restoration (Mei et al., 2016).

## 4 CONCLUSION

In this study, we have provided a draft genome and evolutionary analysis of the water river buffalo from Bangladesh. This study has shed light on the genomic synteny, phylogenetic position and split time among the Artiodactyla order. The integrated water buffalo genome map shows a brief overview of the evolutionary characteristics we have elaborated upon above. In addition, we have presented a usable water buffalo genome which has important practical purposes for economic application to water buffalo‐derived products. Moreover, this will be useful for generating a water buffalo reference genome for data mining, in order to promote the genetic engineering, molecular research, and breeding of buffalo.

## CONFLICT OF INTERESTS

The authors declare that they have no competing interests.

## AUTHORS' CONTRIBUTIONS

QiangGaoand Abdul AwalMintoo designed the study; Tabith M. Awal, M. Moniruzzaman, Mahbub Anam, Quazi Mohammad. Emdadul Huque, and AsmaKhatun performed the experiments; Chunhai Chen, XuanminGuang, Hailin Zhang, Tabith M. Awal, M. Moniruzzaman, MahbubAnam, Xianwei Liang, and Ping Wang analyzed the data; Zhen Zhong, Pengfei Han coordinate and manage the project; M. Moniruzzaman, Tingxian Deng, XuanminGuang, Chunhai Chen, MahbubAnam, and Abdul AwalMintoo wrote the manuscript. All authors read and approved the final manuscript.

## Data Availability

This whole‐genome shotgun project has been deposited at DDBJ/ENA/GenBank under the accession NPZD00000000. The version described in this paper is version NPZD01000000. Raw DNA sequencing reads have been submitted to the NCBI Sequence Read Archive database (SRA488780).
